# Probing the Interactions of Sulfur-Containing Histidine Compounds with Human Gamma-Glutamyl Transpeptidase

**DOI:** 10.3390/md17120650

**Published:** 2019-11-20

**Authors:** Alfonsina Milito, Mariarita Brancaccio, Michael Lisurek, Mariorosario Masullo, Anna Palumbo, Immacolata Castellano

**Affiliations:** 1 Department of Biology and Evolution of Marine Organisms, Stazione Zoologica Anton Dohrn, 80121 Naples, Italy; alfonsina.milito@szn.it (A.M.); mariarita.brancaccio@szn.it (M.B.); palumbo@szn.it (A.P.); 2 Department of Computational Chemistry and Drug Design, Leibniz-Forschungsinstitut für Molekulare Pharmakologie, 13125 Berlin, Germany; lisurek@fmp-berlin.de; 3 Department of Human Movement Sciences and Wellbeing, University of Naples “Parthenope”, 80133 Naples, Italy; mario.masullo@uniparthenope.it

**Keywords:** ovothiol, sulfur-containing histidine, ergothioneine, γ-glutamyl transpeptidase, GGT, marine drugs, docking simulation, enzymatic inhibition, molecular interactions, marine antioxidant

## Abstract

Gamma-glutamyl transpeptidase (GGT) is a cell surface enzyme involved in glutathione metabolism and maintenance of redox homeostasis. High expression of GGT on tumor cells is associated with an increase of cell proliferation and resistance against chemotherapy. GGT inhibitors that have been evaluated in clinical trials are too toxic for human use. We have previously identified ovothiols, 5(Nπ)-methyl-thiohistidines of marine origin, as non-competitive-like inhibitors of GGT that are more potent than the known GGT inhibitor, 6-diazo-5-oxo-l-norleucine (DON), and are not toxic for human embryonic cells. We extended these studies to the desmethylated form of ovothiol, 5-thiohistidine, and confirmed that this ovothiol derivative also acts as a non-competitive-like GGT inhibitor, with a potency comparable to ovothiol. We also found that both 5-thiohistidine derivatives act as reversible GGT inhibitors compared to the irreversible DON. Finally, we probed the interactions of 5-thiohistidines with GGT by docking analysis and compared them with the 2-thiohistidine ergothioneine, the physiological substrate glutathione, and the DON inhibitor. Overall, our results provide new insight for further development of 5-thiohistidine derivatives as therapeutics for GGT-positive tumors.

## 1. Introduction

Sulfur-containing histidine compounds are a class of amino acid derivatives with unique chemical properties, which make them powerful scavengers of radicals and peroxides [1]. Among them, l-ergothioneine (erg) is a natural trimethyl-2-thiohistidine, which was first isolated from rye ergot (*Claviceps purpurea*). To date, the organisms known to be able to synthesize l-ergothioneine are bacteria, more specifically mycobacteria and cyanobacteria [2,3], and fungi of the phyla *Ascomycota*, *Zygomycota* and *Basidiomycota*, including higher edible fungi [4]. Higher organisms, including humans, acquire erg by diet and mainly accumulate it in tissues such as liver, blood, kidneys and heart due to the high affinity of erg to the organic cation transporter 1 [5,6,7]. Ovothiols, 5-thiohistidine derivatives, characterized by a methyl group on the imidazole ring of histidine, are naturally present in marine invertebrates, bacteria and protists [8,9,10,11] and occur either as free amino acids, or as building blocks of complex thioalkaloids or iron chelating pigments [12]. In the free forms, ovothiols can differ in the rate of methylation at the α-amino group of the lateral chain of 5-thiohistidine. Ovothiol A (ovo), methylated only on the imidazole ring, was isolated from the eggs of sea urchins and from ovary, eggs and biological fluids of other marine invertebrates, such as sea stars, and cephalopods [11,12], from some human pathogens, and microalgae [9,10,13]. The ovothiol derivatives B and C can be distinguished by the presence of one or two additional methyl groups at the aminoacidic lateral chain and are quite uncommon in nature [14]. Thanks to the peculiar position of the thiol group in position 5 of the imidazole ring of histidine, they are probably the most acidic thiols among natural products and are endowed with unique redox properties [12,14,15]. In particular, they can play a key role in keeping the cellular redox balance, due to their ability to make redox-exchange with glutathione (GSH) [14,15].

Ovo has been shown to exert anti-inflammatory activity, when administered in its disulfide form in an *in vitro* model of endothelial dysfunction [16], and in an *in vivo* model of liver fibrosis [17]. Moreover, we have previously shown that ovo induces autophagy in a human liver carcinoma cell line, HepG2, and a leukemia cell line, HG3, through the inhibition of human γ-glutamyl transpeptidase (hGGT) [18,19]. The GGT enzyme (EC 2.3.2.2) is localized on the outside of the cell surface, and by cleaving the γ-glutamyl bond of extracellular GSH, allows the cell to use GSH as a source of cysteine for protein synthesis and increase the formation of intracellular GSH [20,21]. Several human tumors, including hepatocellular carcinoma, exhibit high GGT activity, which enhances their resistance to chemotherapy because of the ability of GGT to recycle GSH and sustain uncontrolled cell growth by increasing protein synthesis [22,23,24,25]. Moreover, higher GGT activity is involved in several other pathologies such as liver fibrosis, ischemia/reperfusion-induced renal injury, and asthma [17,26,27]. We have previously demonstrated that sulfur-containing histidine compounds act as non-competitive-like inhibitors of GGT, which are more potent compared to other compounds of chemical synthesis that have been abandoned in clinical trials due to toxicity [19]. In this way, the antioxidant function of ovothiols has a dual nature. Indeed, when ovo enters the cell, it can directly react with peroxides and GSH to regulate redox homeostasis [16], whereas, when interacting with membrane bound GGT, it can indirectly regulate GSH metabolism and redox homeostasis [19].

In detail, GGT catalyzes the cleavage of γ-glutamyl compounds and the transfer of the γ-glutamyl group to an acceptor substrate by a ping-pong mechanism [20,21]. GSH, the most common physiological substrate of GGT, acts as the γ-glutamyl donor in the initial reaction of hydrolysis. In particular, a catalytic Thr (Thr381 in hGGT) within the active site, acts as a nucleophile [28] and attacks the δ-carbon of the glutamate moiety, leading to the formation of a tetrahedral intermediate (γ-glutamyl enzyme complex), stabilized by two conserved glycines (Gly473 and Gly474 in hGGT) [29]. The positioning of the donor substrate inside the active site is helped by hydrogen bonds between the α-carboxyl and the α-amino groups of the glutamate and key neighbor residues (Arg107, Ser451, Ser452 and Asn401 in hGGT) as well as by a salt bridge between the α-amino group of the glutamate and a glutamic acid residue (Glu420 in hGGT) [29]. A salt bridge between Asp423 and Arg107 further stabilizes the glutamate-hGGT complex [29]. Following the first reaction, the cysteinyl–glycine dipeptide is released and cleaved into cysteine and glycine by cell surface dipeptidases, while the departing γ-glutamyl group is transferred from the γ-glutamyl-GGT complex to the second substrate (the acceptor), which can be a molecule of water, in the case of a hydrolysis reaction, or amino acids/dipeptides during the second reaction catalyzed by GGT, called transpeptidation [20,21]. Acceptors bind to the GGT acceptor site through conserved residues in hGGT, including Lys562 and Tyr403 [29].

The most well-known compounds that inhibit GGT include the glutamine analogues Acivicin ((2*S*)-2-amino-2-[(5*S*)-3-chloro-4,5-dihydro-1,2-oxazol-5-yl]acetic acid), DON (6-diazo-5-oxo-l-norleucine), and Azaserine (*O*-diazoacetyl-l-serine) [30,31]. However, most of these glutamine analogues have proved to be toxic, due to their inhibition of glutamine transferases and their interference with the glutamate–glutamine cycle, leading to innumerable side effects on pathways related to the recycling of the γ-aminobutyric acid neurotransmitter [32].

Sulfur derivatives of l-glutamic acid and γ-(monophenyl)phosphono glutamate analogues also function as inhibitors of GGT and are less toxic compared to glutamine analogues [33,34,35,36]. For example, a butanoic acid derivative (GGsTop) was found to be effective for the treatment of ischemia/reperfusion-induced renal injury, asthma, and oral mucositis [26,27,37]. Moreover, a class of uncompetitive inhibitors, which bind the acceptor site and are less toxic than glutamine analogues, have been developed, but no further in vitro and in vivo studies have confirmed their therapeutic potential [38].

Intense efforts are continuously devoted to characterize new inhibitors of GGT due to their therapeutic potential in the treatment of GGT-dependent pathologies. In particular, most of the known GGT inhibitors act by an irreversible mechanism often leading to off-targets effects. Therefore, the search for reversible inhibitors represents an important challenge, due to several advantages for therapeutic use, including the higher degree of modulation and lower toxicity. In this study, we aim to characterize the interactions of the novel sulfur-containing histidine inhibitors, ovo and des-methylated 5-thiohistidine (5-thio), with hGGT in order to predict a mechanism of inhibition. Molecular docking simulations among these compounds and hGGT were compared with the physiological substrate GSH and the DON inhibitor.

## 2. Results

### 2.1. Kinetics of GGT Inhibition by 5-Thiohistidine 

We have previously demonstrated that ovo inhibits GGT by a non-competitive mechanism [19]. To confirm that the des-methylated form 5-thio also acts as a GGT inhibitor, enzymatic assays were carried out using equine kidney GGT (eqGGT with high % of identity with hGGT), maintaining fixed and saturating concentrations of γ-glutamyl-para-nitroanilide (GpNa) as a donor substrate and glycyl-glycine (GlyGly) as the acceptor, and varying the concentrations of 5-thio, used in its disulfide form. The analysis of the residual GGT activity in the presence of 5-thio demonstrated that 50% of GGT inhibition was obtained at 15 µM, a value very similar to the 16 µM determined for ovo, and significantly lower than that of 282 µM for DON, and 297 µM for erg [19]. The addition of DTT (dithiothreitol) did not induce any GGT inhibitory action, thus excluding the possibility that the observed inhibitory effect was due to the intrinsic ability of an unspecific thiol to reduce cysteine or disulfides.

**Figure 1:**
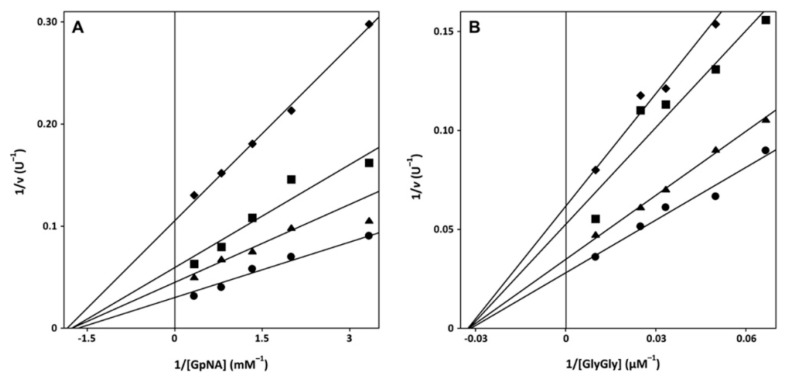
Kinetic analysis of GGT inhibition. EqGGT activity was assayed as reported in Materials and Methods in the presence of 40 mM GlyGly and the indicated concentrations of GpNA (**A**) or in the presence of 3 mM GpNA and the indicated concentrations of GlyGly (**B**). Double reciprocal Linweaver–Burk plots were obtained in the absence (closed circle) or in the presence of 5 (closed triangles), 10 (closed squares) or 20 (closed diamonds) µM 5-thio (Panels A, B).

To unravel the mechanism of 5-thio driven inhibition of GGT, kinetics of eqGGT activity were analyzed, in the absence or in the presence of the inhibitor, varying the concentration of the donor substrate with fixed concentration of the acceptor, and vice versa. The effect of different concentrations of 5-thio, ranging from 5 to 20 µM, on the kinetics of eqGGT is shown in the representative Lineweaver-Burk graphs reported in Figure 1. The behavior of 5-thio inhibition accounted for a non-competitive-like inhibition, as shown from the finding that the extrapolation of the fitting on the x axis approached very similar *K*_m_ values, with a decreasing *V_max_*. These data suggested that 5-thio has the same affinity for the free enzyme and the covalent *E-*γ-glutamyl complex, both when GpNA binds as donor substrate and GlyGly binds as acceptor, similar to what happened for the kinetic behaviors in the presence of ovo and erg [19]. The apparent *K*_i_ values obtained from *K*_m_, calculated by nonlinear fitting in the Michaelis–Menten equation, were 17 µM ± 1.5, when varying GlyGly concentrations, and 7 µM ± 1.7 when varying GpNA concentrations. These values indicated that 5-thio behaves similarly to ovo (*K*_i_ values: 21 µM ± 7, when varying GlyGly concentrations, and 7 µM ± 1 when varying GpNA concentrations), being a more potent GGT inhibitor compared to erg and DON [19]. 

### 2.2. Mechanism of Inactivation of GGT by Sulfur-Containing Compounds and Cytotoxicity 

**Figure 2:**
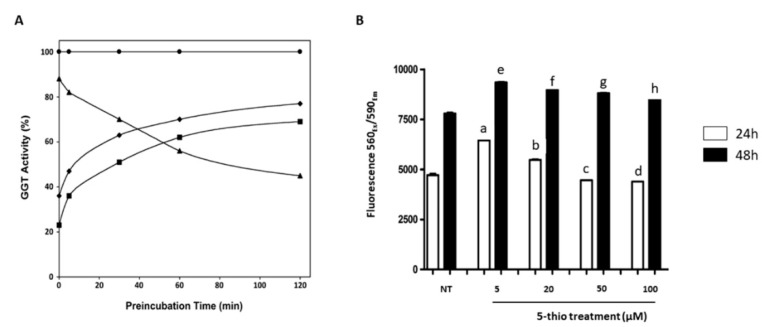
Inhibition of equine gamma-glutamyl transpeptidase (eqGGT) by 5-thiohistidines and cytotoxicity assay of 5-thio on HEK 293 cells. (**A**) EqGGT was preincubated at 37 °C with 400 µM of DON (closed triangles), 20 µM of 5-thio (closed diamonds), 20 µM of ovo (closed squares), or in the absence of compounds (closed circle) for the indicated times. Immediately following the pre-incubation, the mix was assayed for GGT activity as reported in the Materials and Methods section. Experiments were performed in quadruplicate and data were reported as percentage of enzyme activity in the presence of the inhibitors with respect to the enzyme without the inhibitor. (**B**) HEK 293 cells were treated for 24 and 48 h with 5-thio at the indicated concentrations, and cell viability was measured by a resazurin-based assay (*n* = 3). Data were reported as units of fluorescence ± SD. a (*p* ≤ 0.0001); b (*p* = 0.0014); c (*p* = 0.0448); d (*p* = 0.0218) represent significance compared to NT (not treated) at 24 h; e (*p* ≤ 0.0001); f (*p* ≤ 0.0001); g (*p* = 0.0001); h (*p* = 0.0045) represent significance compared to NT (not treated) at 48 h.

The reversibility of the GGT inhibition was studied by pre-incubating eqGGT with ovo, 5-thio and DON for 2 hours and the residual enzymatic activity was measured at different time points after dilution of the pre-incubation mix in the reaction buffer. The kinetics of the pre-treatments indicated that DON acts as an irreversible inhibitor, and then as an inactivator of GGT, because, after dilution, the activity was not restored and it decreased with the incubation time (Figure 2A). An opposite trend was instead observed for 5-thio and ovo, for which the activity was restored in a time dependent manner, after dilution (Figure 2A). This finding indicates a reversible inhibition mechanism for 5-thio and ovo. The addition of DDT to the eqGGT-inhibitors reaction mix did not significantly affect GGT inhibition (Table 1), indicating that the inhibiting species are indeed the disulfide form of ovo and 5-thio.

We also tested 5-thio for toxicity on human kidney embryonic HEK293 cells, as previously done for ovo [19]. In particular, 5-thio was not toxic until 48 h treatment, even showing a slight increase of cell proliferation at low doses, likely due to a hormetic effect (Figure 2B, Table S1 for raw data).

### 2.3. Docking Studies 

**Figure 3:**
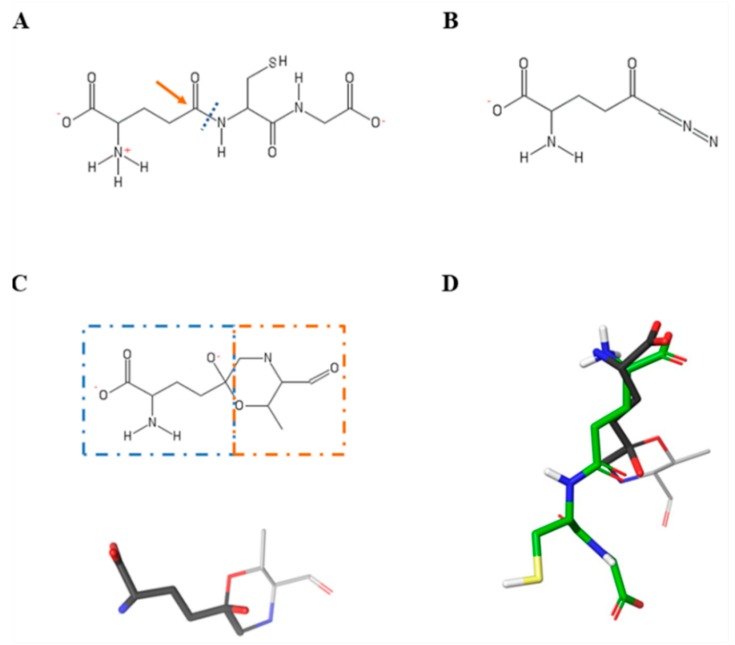
GSH and DON structures and superimposition of their docked poses. (**A**) 2D structure of GSH, in which the γ-glutamyl bond (blue dashed line) is cleaved following a nucleophile attack (orange arrow) to the δ-carbon of the glutamate moiety. (**B**) 2D structure of DON. (**C**) Product of covalent interaction between DON and Thr381 of hGGT both in 2D (on top) and 3D (on bottom) representation. In the 2D structure, dashed lines divide the DON (blue box) from Thr381 (orange box) in the DON–enzyme complex. In the DON 3D structure, carbon atoms are colored grey, oxygen red, nitrogen blue. (**D**) Superimposition of docked poses of GSH and DON into the glutamyl-binding pocket of hGGT. In the GSH 3D structure, carbon atoms are colored green, oxygen red, nitrogen blue, sulfur yellow, polar hydrogens white. Non polar hydrogens are hidden.

The interactions between thiohistidines, i.e., ovo, 5-thio and erg, and key residues within the active site of hGGT were investigated by molecular docking, using the crystal structure of hGGT in complex with GGsTop (4ZBK.pdb), in which the inhibitor was removed. In order to assess the reliability of our method of analysis, we first docked GSH and DON, for which the bound-GGT crystal structure is already known (4GDX.pdb, 5V4Q.pdb). The results of docking analysis showed that the interactions of GSH and DON in the hGGT active site overlapped, in particular for the amino and carboxyl groups of the glutamate moiety (Figure 3).

**Figure 4:**
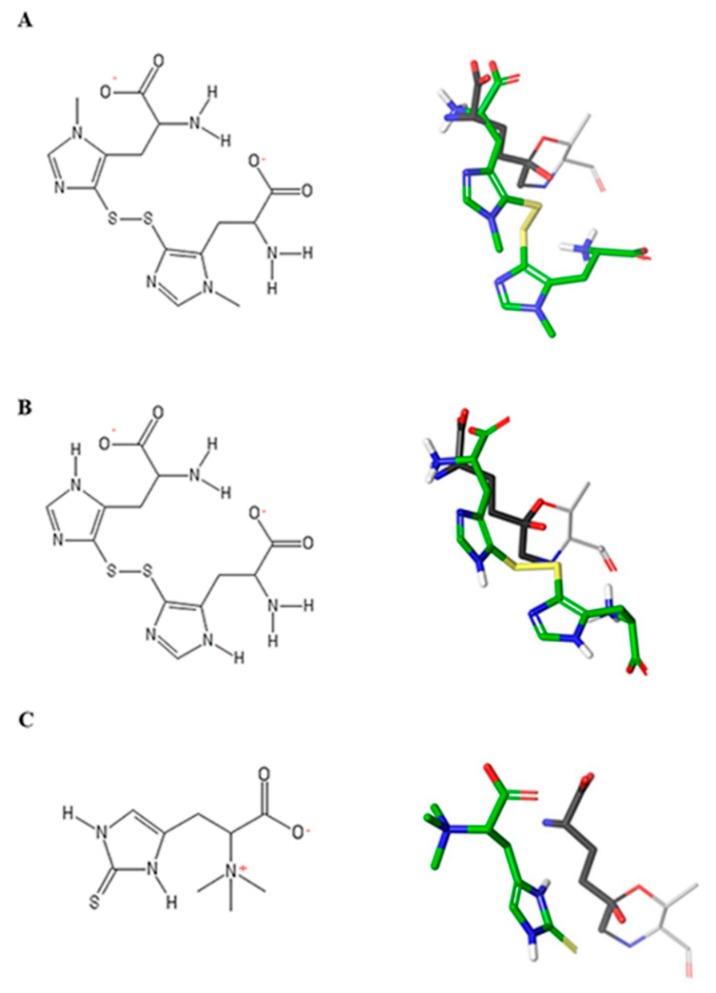
Structures of thiohistidines and superimposition of their docked poses with DON. (**A**) 2D structure of ovo in disulfide form and 3D structure of ovo in disulfide form superimposed with DON. (**B**) 2D structure of 5-thio in disulfide form and 3D structure of ovo in disulfide form superimposed with DON. (**C**) 2D structure of ergothioneine and 3D structure of erg superimposed with DON. In the DON 3D structure, carbon atoms are colored grey, oxygen red, nitrogen blue. In the thiohistidines 3D structures, carbon atoms are colored green, oxygen red, nitrogen blue, sulfur yellow, polar hydrogens white. Non polar hydrogens are hidden.

Then, thiohistidine compounds were docked into the active site of hGGT in their most stable forms. Both ovo and 5-thio were docked in their disulfide form and the resulted poses overlapped with the one of DON (Figure 4A,B). Erg was docked in its thione form and the prediction showed that its pose did not overlap with DON (Figure 4C).

**Figure 5:**
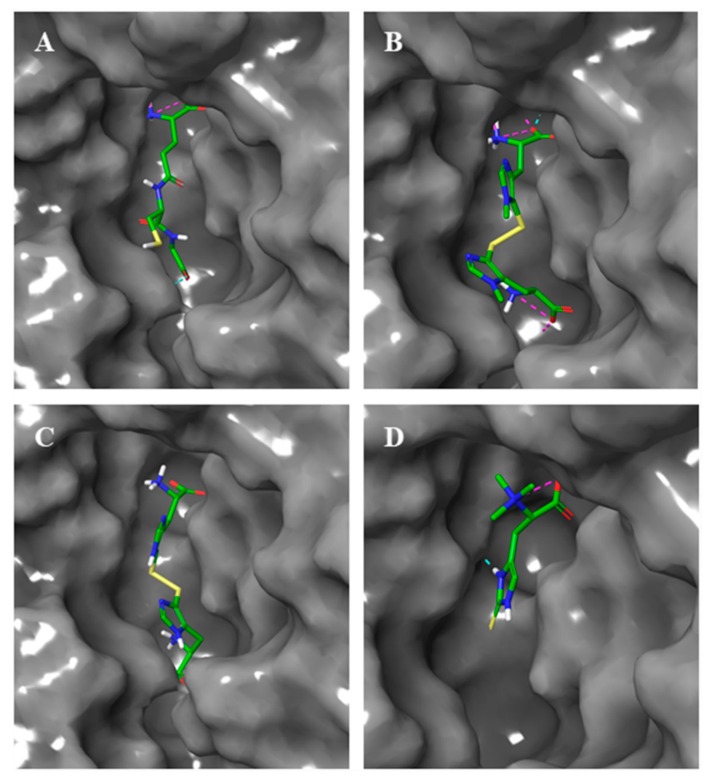
GGT surface view of docking simulations. GSH was docked into the GGT glutamyl binding pocket (**A**). The same was done for ovo (**B**), 5-thio (**C**) and erg (**D**). Carbon atoms of ligands are colored green, oxygen red, nitrogen blue, sulfur yellow, polar hydrogens white. Non polar hydrogens are hidden.

Docking simulations suggested that, as for GSH (Figure 5A), all thiohistidine compounds are able to enter the active site of GGT (Figure 5B–D). However, while ovo and 5-thio, in their disulfide form, occupied both donor and acceptor sites inside the catalytic pocket, the corresponding reduced forms and erg were predicted to place only into the donor substrate site, due to the smaller structure (Supplementary Figure S1 and Figure 5D). 

**Figure 6:**
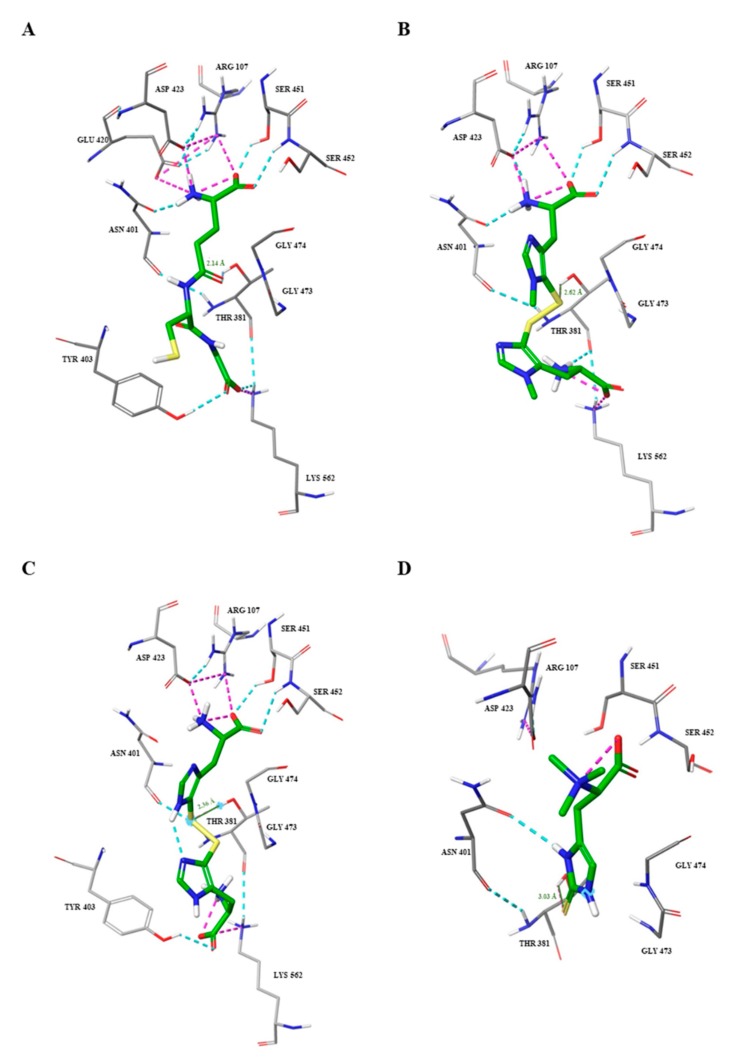
Molecular interactions of GSH and thiohistidine compounds within the hGGT active site. GSH (**A**); ovo (**B**); 5-thio (**C**); erg (**D**). Carbon atoms of the active site residues involved in the interactions are colored light grey, compared to carbon atoms of the ligands which are colored green. Oxygen atoms are colored red, nitrogen blue, sulfur yellow, polar hydrogens white. Non polar hydrogens are hidden. Dashed blue and magenta lines represent hydrogen bonds and salt bridges, respectively. Molecular distance between the *S*- of thiohistidines and OH– of Thr381 are written in orange and indicated by continued orange lines.

In particular, the simulated interactions between ovo and the active site of GGT were mediated by several hydrogen bonds and salt bridges with the key residues involved in the stabilization of the tetrahedral intermediate in the donor site (Asp423, Arg107, Ser451, Ser452, Asn401), as well as with an amino acid residue of the acceptor site (Lys562), perfectly mimicking the interactions occurring with GSH (Figure 6A,B). The same interactions occurred between 5-thio and the active site of hGGT with the exception of an additional hydrogen bond between the carboxyl group of the lateral chain of histidine and the Tyr403 in the acceptor site (Figure 6C). This is probably due to the absence of the methyl group on the imidazole ring of histidine and the lower consequent hindrance. Erg was placed in the active site, but it interacted only with Asn401 by the formation of a hydrogen bond with the nitrogen of the imidazole ring of erg (Figure 6D). Moreover, the molecular distance between the sulfur atom of the thiohistidines and the catalytic –OH of Thr381 was calculated to be shorter for ovo and 5-thio (2.62 Å and 2.36 Å, respectively) compared to erg (3.03 Å) (Figure 6A–D). The simulated interactions of the reduced forms of ovo and 5-thio with hGGT involved only residues in the donor binding site (Supplementary Figure S1).

## 3. Discussion and Conclusions

GGT represents an important enzymatic target for the therapy of a wide variety of pathologies, such as tumors [22,23,24,25], liver fibrosis [17], ischemia/reperfusion-induced renal injury and asthma [26,27], thus the development of inhibitors of this enzyme with high specificity and low toxicity represents a booming field of research. Glutamine analogues have been developed, acting as irreversible competitive inhibitors due to their binding affinity for the donor site, but most of them have been abandoned in clinical trials due to toxicity [30,31,32]. Glutamic acid/glutamate derivatives have been proven to provide a valid therapeutic alternative, as these irreversible GGT inhibitors are more selective and less toxic [33,34,35,36,37]. Finally, a novel class of uncompetitive inhibitors has also been identified, whose action is displayed by binding to the acceptor site of the enzyme, thus resulting in less toxicity compared to the glutamine analogues [38]. In general, uncompetitive inhibitors are more advantageous for in vivo applications because they are more efficient in inhibition as the enzyme substrate rises in concentration, differently from competitive inhibitors, which become less potent as the substrate concentration increases [39]. Moreover, a reversible mode of action for GGT inhibitors could provide the additional advantage of a rapid inhibition rate [40], as well as being easier to modulate in vivo, and thus preserving physiological GGT activity and resulting in lower toxicity.

We have previously identified a novel class of non-competitive-like GGT inhibitors, i.e., methyl-5-thiohistidine of marine origin (ovothiols), as an alternative therapeutic strategy for GGT-dependent diseases [16,17,18,19,41].

Here, we demonstrated that the des-methylated form of ovothiol (5-thio) also acts as a non-competitive-like inhibitor of GGT with *Ki* values very similar to ovo, and it is non-toxic towards human kidney embryonic cells. Moreover, we proved that both 5-thiohistidines, ovo and 5-thio, act as reversible GGT inhibitors compared to DON, which forms a stable and irreversible DON–GGT complex, consisting of a six-membered ring involving the catalytic Thr381 [31]. These data suggest that 5-thiohistidine derivatives can be finely modulated in in vitro and in vivo studies, thanks to their reversible mode of action. In particular, 5-thio has the advantage of being efficiently obtained by chemical synthesis [42]; hence, it can be considered a valid alternative to ovo, whose synthesis is tricky. Moreover, by docking analysis, we predicted that disulfide forms of ovo and 5-thio enter the hGGT binding pocket, and interact with key residues in the donor and the acceptor binding sites, which are the same as those involved in the formation of the tetrahedral intermediate of the physiological substrate GSH, whereas erg is far from establishing the same interactions. This may explain the stronger inhibitory power of 5-thiohistidine disulfides in inhibiting hGGT compared to erg [19]. The finding that both ovo and 5-thio in disulfide also interact with one or two residues in the acceptor site, may explain the stronger inhibitory activity of both molecules compared to DON and the reduced forms of ovo and 5-thio, whose interactions involve only residues in the donor binding site. Considering the small distance between the –OH group of the catalytic Thr381 and the disulfide bond of ovo (2.62 Å) and 5-thio (2.36 Å), we hypothesize that GGT inhibition involves initial binding of the disulfide form of these molecules, with the consequent release of the reduced form and the formation of an ovoS–enzyme complex. This could explain the recovery of GGT enzymatic activity following pre-incubation with ovo and 5-thio compared to the inactivated DON–enzyme complex. Further studies will be necessary to confirm our proposed mechanism of inhibition. In particular, site-directed mutagenesis of hGGT followed by enzyme kinetics studies will provide the proof for the involvement of the key residues highlighted in this work in the inhibiting activity of 5-thiohistidines. In addition, studies aimed at unveiling the crystallographic structure of hGGT bound with 5-thiohistidine derivatives will shed new light on the mechanism of inhibition of these compounds for their potential use as new marine drugs to treat GGT-positive pathologies like liver fibrosis, some types of cancer, asthma, and ischemia/reperfusion-induced renal injury.

Finally, the recent discovery that ovothiol biosynthetic pathway is more widespread in nature than previously thought [43] suggests that ovothiol derivatives with unexplored chemical and biological activities may be found for the development of novel marine drugs in the next future.

## 4. Materials and Methods 

### 4.1. GGT Activity, Kinetic Studies and Data Analysis

GGT from equine kidney (EqGGT) was purchased by Sigma-Aldrich. The compound 5-thio was produced according to Daunay [42], and kindly provided by Prof. Florian Peter Seebeck, University of Basel. Ovo was purified by sea urchin eggs, as previously described [18]. GGT activity was determined by a colorimetric test. The assay buffer contained 100 mM Na_2_HPO_4_, pH 7.4, with 3.2 mM KCl, 1.8 mM KH_2_PO_4_, and 27.5 mM NaCl 100 mM (Phosphate Buffered Saline—PBS). Each reaction contained 3 mM of γ-glutamyl-para-nitroanilide (GpNA) as a donor substrate and 40 mM glycyl glycine (GlyGly) as an acceptor substrate. The *p*-nitroaniline formation was continuously monitored at room temperature by reading the absorbance at 405 nm using a Bio-Rad 680 microplate reader with Microplate Manager 5.2 (Bio-Rad) software. One unit of GGT activity was defined as the amount of GGT that released 1 μmol of p-nitroaniline/min at room temperature. To carry out kinetics studies, the concentrations of the substrate GpNA and the acceptor GlyGly were varied as indicated in the figure legends. Aliquots of 0.5–1 µL GGT (1 mg/mL) were added to start the reaction, which was time-monitored as described above [19] using a Cary100 spectrophotometer (Agilent, Santa Clara, CA, USA). The initial velocity of the reaction was derived from the linear part of the kinetic. Data were nonlinearly fitted in the Michaelis–Menten equation using the Kaleidagraph™ 4.1 software (Synergy). 

The apparent inhibition constant (*K*_i_) was calculated using the equation:

*V*_max_′ = *V*_max_/(1 + [*I*]/*K*_i_)

where *V*_max_ and *V*_max_′ represent the maximum velocity in the absence or in the presence of the [*I*] concentration of the inhibitor. The values of the apparent *K*_i_ obtained at each inhibitor concentration were averaged to obtain a unique value of apparent *K*_i_. 

Kinetic parameters and their corresponding standard errors were evaluated using a simple weighting method (*t*-Student test).

### 4.2. Time-Dependent Inhibition of hGGT by Thiohistidine Compounds 

The reversibility of the inhibition was assessed using a dilution method described previously [44]. Briefly, the enzyme eqGGT 7 µM was pre-incubated at 37 °C for different time points with the inhibitors or with DTT, as a control, in a total volume of 60 µL of water. After a 50-fold dilution of the incubation mixture, the residual enzyme activity was measured. The concentrations of the inhibitors in the preincubation mix were 400 µM of DON, 20 µM of ovo and 20 µM of 5-thio, respectively. To study the effect of the reduced forms of ovo and 5-thio, DTT was added at 5 mM for the time indicated. The mix assay containing 1× PBS, 1 mM of GpNA and 20 mM of GlyGly, and the amount of product released was monitored as described above. The percentage of recovered enzyme activity was referred to that measured for the enzyme pretreated under identical experimental conditions in the absence of inhibitors.

### 4.3. Cytotoxicity Assays

For cytotoxicity assays, HEK 293 cells were plated in 384-multiwell plates (2 × 10^3^ cell/well) by a pipetting robot (Freedom Evo-2 200 Liquids Handler, Tecan, Männedorf, Switzerland) and treated with 5-thio, at the indicated concentrations for 24–48 h. Cytotoxicity was assessed by resazurin-based assays (CellTiter-Blue^®^ Cell Viability Assay, Promega, Madison, WI, USA) according to the manufacturer’s recommendations using a Spark^®^ multimode microplate reader (TECAN). Experiments were performed in triplicate. Data were expressed as units of fluorescence 560_Ex_/590_Em_ nm.

### 4.4. Molecular Docking of Thiohistidines into the GGT Active Site 

The 2D/3D structures of thiohistidines, including ovo, 5-thio and erg, and the ones of the natural substrate of the GGT enzyme, i.e., glutathione, and of the commercial GGT inhibitor, DON, were constructed using PubChem Sketcher V2.4 and SYBYL-X 2.1 software (https://sybyl-x.software.informer.com/2.1/). The crystal structure of human GGT in complex with the GGsTop inhibitor (4ZBK.pdb) was chosen for the study and was derived from the Protein Data Bank (https://www.ebi.ac.uk/pdbe/). Before the docking, water molecules and the inhibitor GGsTop were removed from the protein structure. The hits were docked into the active site of GGT by the use of Glide within Schrödinger-Maestro (Glide; Schrödinger Release 2016—4: Glide, Schrödinger, LLC, New York, NY, USA, 2016) [45,46,47] and the use of three H-bond constraints in the setting conditions (Ser452, Asp423, Asn401). The resulting docking poses were inspected visually and the best scored pose with the typical hydrogen network observed in the crystal structure was chosen.

## Figures and Tables

**Table 1:** Time-dependent inhibition of eqGGT activity by 5-thiohistidines ± DTT. The % of residual GGT activity after the incubation with 5 mM of DTT, 20 µM of ovo, 20 µM of 5-thio, ovo/ 5-thio ± DTT at the same concentrations, or in absence of the compounds at 0, 30 and 60 min, is reported.

Time (min)	eqGGT	DTT	ovo	5-thio	DTT + ovo	DTT + 5-thio
0	100%	93%	39%	33%	94%	86%
30	100%	89%	60%	47%	83%	71%
60	100%	72%	72%	59%	73%	60%

## Supplementary Materials

The following are available online at https://www.mdpi.com/1660-3397/17/12/650/s1, Figure S1: Molecular interactions of the reduced ovo and 5-thio within hGGT active site.
