# Supplementary material for: Probing the Interactions of Sulfur-Containing Histidine Compounds with Human Gamma-Glutamyl Transpeptidase
Source: Mar Drugs. 2019 Nov 20;17(12):650. doi: 10.3390/md17120650 (PMC6949936; doi:10.3390/md17120650)
Supplement: Supplementary file 1 [file marinedrugs-17-00650-s001.pdf]

Probing the interactions of sulfur-containing histidine compounds with human gamma-glutamyl transpeptidase

Alfonsina Milito[1], Mariarita Brancaccio[1], Michael Lisurek[2], Mariorosario Masullo[3], Anna Palumbo[1], Immacolata Castellano[1,*]

[1] Dept. of Biology and Evolution of Marine Organisms, Stazione Zoologica Anton Dohrn, Naples (Italy)
[2] Dept. of Computational Chemistry and Drug Design, Leibniz-Forschungsinstitut für Molekulare Pharmakologie, Berlin (Germany)
[3] Dept. of Human Movement Sciences and Wellbeing, University of Naples "Parthenope", Naples (Italy)

Table S1. Cytotoxicity assay on HEK 293 cells. Cell vitality raw data were reported as units of fluorescence following the treatment with 5–thio for 24 and 48h (n=3).

Condition	Average ± SD
NT 24h	4717 ± 153
5-thio 5 µM	6452 ± 108
5-thio 20 µM	5470 ± 71
5-thio 50 µM	4490 ± 22
5-thio 100 µM	4395 ± 5
NT 48h	7794 ± 105
5-thio 5 µM	9353 ± 76
5-thio 20 µM	8969 ± 20
5-thio 50 µM	8869 ± 52
5-thio 100 µM	8557 ± 205

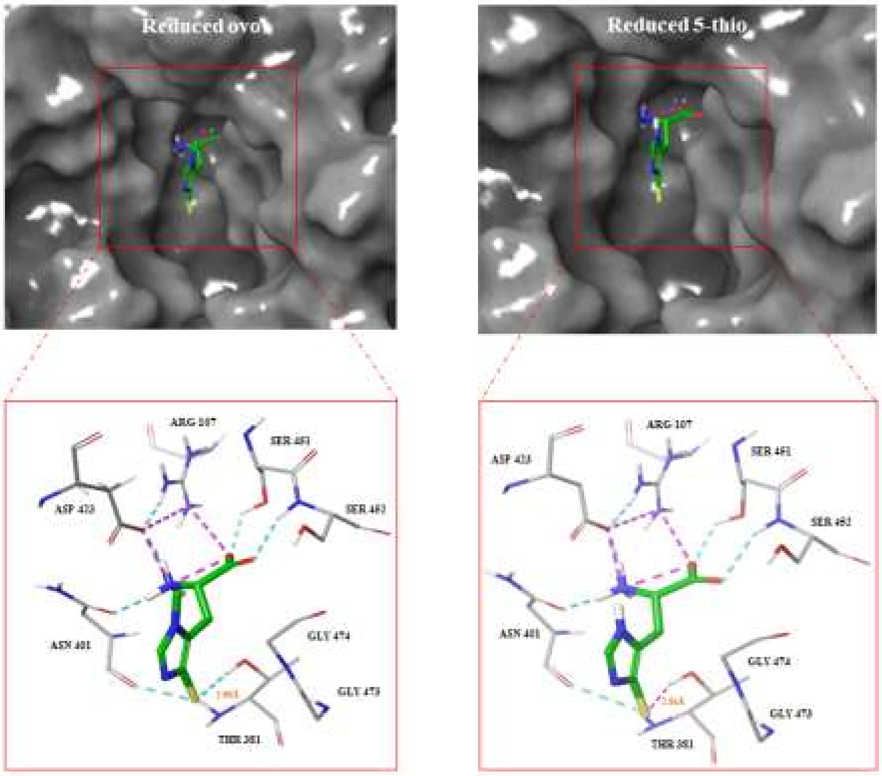

Figure S1. Molecular interactions of the reduced ovo and 5-thio within hGGT active site. On top: hGGT surface view from docking simulations; on bottom: interactions among ovo/5-thio and key residues in the active site of hGGT. Carbon atoms of the active site residues are *colored light grey*, compared to carbon atoms of the ligands which are *colored green*. Oxygen atoms are *colored red*, nitrogen *blue*, sulfur *yellow*, polar hydrogens *white*. Non polar hydrogens were hidden. Dashed blue and magenta lines represent hydrogen bonds and salt bridges, respectively. Molecular distance between ovo/5-thio and OH- of Thr381 are written in orange.
